# Protein (multi-)location prediction: using location inter-dependencies in a probabilistic framework

**DOI:** 10.1186/1748-7188-9-8

**Published:** 2014-03-19

**Authors:** Ramanuja Simha, Hagit Shatkay

**Affiliations:** 1 Department of Computer and Information Sciences, University of Delaware, Newark DE, USA; 2 School of Computing, Queen’s University, Kingston ON, Canada; 3 Center for Bioinformatics and Computational Biology, DBI, University of Delaware, Newark DE, USA

## Abstract

**Motivation:**

Knowing the location of a protein within the cell is important for understanding its function, role in biological processes, and potential use as a drug target. Much progress has been made in developing computational methods that predict single locations for proteins. Most such methods are based on the over-simplifying assumption that proteins localize to a single location. However, it has been shown that proteins localize to multiple locations. While a few recent systems attempt to predict multiple locations of proteins, their performance leaves much room for improvement. Moreover, they typically treat locations as independent and do not attempt to utilize possible inter-dependencies among locations. Our hypothesis is that directly incorporating inter-dependencies among locations into both the classifier-learning and the prediction process can improve location prediction performance.

**Results:**

We present a new method and a preliminary system we have developed that directly incorporates inter-dependencies among locations into the location-prediction process of multiply-localized proteins. Our method is based on a collection of Bayesian network classifiers, where each classifier is used to predict a single location. Learning the structure of each Bayesian network classifier takes into account inter-dependencies among locations, and the prediction process uses estimates involving multiple locations. We evaluate our system on a dataset of single- and multi-localized proteins (the most comprehensive protein multi-localization dataset currently available, derived from the DBMLoc dataset). Our results, obtained by incorporating inter-dependencies, are significantly higher than those obtained by classifiers that do not use inter-dependencies. The performance of our system on multi-localized proteins is comparable to a top performing system (YLoc^+^), without being restricted only to location-combinations present in the training set.

## Background

Knowing the location of a protein within the cell is essential for understanding its function, its role in biological processes, as well as its potential role as a drug target [1]. Experimental methods for protein localization such as those based on mass spectrometry [2] or green fluorescence detection [3], although often used in practice, are time consuming and typically not cost-effective for high-throughput localization. Hence, much ongoing effort has been put into developing high-throughput computational methods [4-8] to obtain proteome-wide location predictions.

Over the last decade, there has been significant progress in the development of computational methods that predict a *single* location per protein. The focus on single-location prediction is driven both by the data available in public databases such as UniProt [9], where proteins are typically assigned a single location, as well as by an (over-)simplifying assumption that proteins indeed localize to a single location. However, proteins do localize to multiple compartments within the cell [10-13], and translocate from one location to another [14]. Identifying the mutiple locations of a protein is important because translocation can serve some unique functions. For instance, GLUT4, an insulin-regulated glucose transporter, which is stored in the intracellular vesicles of adipocytes, translocates to the plasma membrane in response to insulin [15,16]. As proteins do not localize at random and translocations happen between designated inter-dependent locations, we hypothesize that modeling such inter-dependencies can help in predicting protein locations. Thus, we aim to identify associations or *inter-dependencies* among locations and leverage them in the process of predicting locations for proteins.

Several methods have been recently suggested for predicting multiple locations for proteins. For instance, King and Guda introduced ngLOC [17], which uses a naïve Bayes classifier (see e.g. [18]) to obtain a probability distribution over locations for a query protein, where each location probability is computed *independently*. Each protein is represented as an *n*-gram constructed based on its amino acid sequence. For a query protein, estimates of the conditional probabilities of the protein to be localized to each location, given its amino acid sequence, are determined. Using the estimates of the two most probable locations, a *multi-localized confidence score* is computed as a measure of the likelihood of the protein to be localized to both locations; if the score is above a certain threshold, the protein is predicted to be assigned to both locations. This method is limited to proteins that are localized to at most two locations.

Li et al. [19] construct multiple binary classifiers, where each binary classifier distinguishes between a pair of locations (one vs. one). Each binary classifier consists of an ensemble of *k*-nearest neighbors (*k*-NN) (see e.g. [20]) and Support Vector Machines (SVMs) (see e.g. [20,21]). The protein representation used in the binary classifiers is based on sequence-derived features (e.g. amino acid composition) and gene ontology (GO) terms. The predictions from all the classifiers are combined to obtain a score for each location. A query protein is assigned to the location with the highest score. If multiple locations have the same highest score, a multi-location prediction is made and all the locations sharing the highest score are predicted for the protein.

Several methods use variations of *k*-NN to predict multiple locations for proteins. WoLF PSORT [22,23] uses *k*-NN with a distance measure that combines Euclidean and Manhattan distances, Euk-mPLoc [24] uses an ensemble of *k*-NN, and iLoc-Euk [25] uses a multi-label *k*-NN classifier. Both WoLF PSORT and Euk-mPLoc represent proteins based on sequence-derived features, while Euk-mPLoc also uses relevant GO terms. Proteins in iLoc-Euk are represented either using relevant GO terms or using features that aim to capture the likely substitutions along the proteins’ amino acid sequences over time. Given a query protein, WoLF PSORT assigns it to the location-combination that is most common among the protein’s *k* nearest neighbors, thus limiting the method to predicting location-combinations present in the training set. The two systems iLoc-Euk and Euk-mPLoc both compute a score for each location, based on the query protein. iLoc-Euk assigns the protein to the locations having the highest scores; the number of locations assigned is the same as that associated with the nearest neighbor protein in the dataset. Euk-mPLoc assigns the query protein to locations whose score lies within a certain deviation from the highest score. iLoc-Euk was not extensively tested against existing multi-location predictors. Moreover, to achieve the reported level of performance, iLoc-Euk strongly relies on features that are only available for proteins that are already annotated. The performance of Euk-mPLoc was evaluated using an extensive dataset [26] and is the lowest among current multi-location predictors. Methods similar to iLoc-Euk were proposed for localizing subsets of eukaryotic proteins [27,28], virus proteins [29], and bacterial proteins [30,31]. Several domain-specific systems using the same ideas have been introduced by the same group (Euk-mPLoc 2.0 [32], Hum-mPLoc 2.0 [33], Plant-mPLoc [34], and Virus-mPLoc [35]).

In contrast to the approaches listed above that use feature-based similarity, KnowPred_site_[36] uses sequence-based similarity to construct a collection of location-annotated peptide fragments and predict multiple locations for proteins. The collection is built by extracting for each protein in the training dataset peptide fragments from its sequence and from sequences similar to its sequence; each fragment is annotated with the protein’s locations. The peptide fragments for a query protein are obtained in a similar manner, and the system uses the location annotations of matching peptide fragments in the collection to compute a score for each location. Using the two highest location scores, a multi-localized confidence score is computed to determine if the protein is multi-localized. This method is restricted to predictions of at most two locations for a protein (similar to that seen earlier for ngLOC [17]).

Notably, none of the above methods for predicting multiple locations utilizes inter-dependencies among locations in the prediction process. All the above models independently predict each single location and thus do not take into account predictions for other locations.

Recent work by He et al. [37] attempts to take advantage of *correlation* among locations when predicting multiple locations of proteins. As part of their classifier training process, an imbalanced multi-modal multi-label learning (which they denote IMMML) classifier attempts to learn a correlation measure between pairs of locations that is later used to make the predictions. The protein representation used in IMMML is based on sequence-derived features (amino acid composition and pseudo-amino acid composition) and gene ontology (GO) terms. While this system takes into account a simple type of dependency among locations, namely pair-wise correlation between locations, it does not account for any more complex inter-dependencies. Furthermore, this system was not tested on any extensive protein multi-localization dataset.

YLoc^+^[26], a comprehensive system for protein location prediction, uses a naïve Bayes classifier (see e.g. [18]) and captures protein localization to multiple locations by explicitly *introducing a new class for each combination of locations supported by the training set* (i.e. having proteins localized to the combination). Thus, each prediction performed by the naïve Bayes classifier can assign a protein to only those combinations of locations included in the training data. To produce its output, YLoc^+^ transforms the prediction into a multinomial distribution over the individual locations. We also note that as the number of possible location-combinations is exponential in the number of locations, training the naïve Bayes classifier in this manner does not provide a practical model in the general case of multi-localized proteins, beyond the training set. The performance of YLoc^+^ was evaluated using an extensive dataset [26] and is the highest among current multi-location predictors.

In this paper, we present a new method that directly models inter-dependencies among locations and incorporates them into the process of predicting locations for proteins. Our system is based on a collection of Bayesian network classifiers (see e.g. [38]). Each Bayesian Network (BN) related to each classifier corresponds to a single location *L*. Each such network is used to assign a conditional probability for a protein to be found at location *L*, given both the protein’s features and *information regarding the protein’s other possible locations*. Learning each BN involves learning the dependencies among the other locations that are primarily related to proteins localizing to location *L*. For each Bayesian network classifier, its corresponding BN is learnt with the goal to improve the classifier’s prediction quality. The formulation of multi-location prediction as classification via Bayesian networks, as well as the network model are presented in the next section. Notably, our system does not assume that *all* proteins it classifies are multi-localized, but rather more realistically, that proteins may be assigned to one or more locations.

We train and test our preliminary system on a dataset containing single- and multi-localized proteins previously used in the development and testing of the YLoc^+^ system [26], which includes the most comprehensive collection of multi-localized proteins currently available, derived from the DBMLoc dataset [11]. As done in other studies [7,8,26,39], we use multiple runs of 5-fold cross-validation. The results clearly demonstrate the advantage of using location inter-dependencies. The *F*_1_ score of 81% and overall accuracy of 76% obtained by incorporating inter-dependencies are significantly higher than the corresponding values obtained by classifiers that do not use inter-dependencies. Also, while our system retains a level of performance comparable to that of YLoc^+^ on the same dataset, we note that unlike YLoc^+^, by training the individual classifiers to predict individual - although inter-dependent - locations, the training of our system is not restricted to only those combinations of locations present in the dataset, thus our system is generalizable to multi-locations beyond those included in the training set.

The rest of the paper proceeds as follows: The next section formulates the problem of protein subcellular multi-location prediction and briefly provides background on Bayesian networks and relevant notations. The Methods section discusses the structure, parameters, and inter-dependencies comprising our Bayesian network collection, and introduces the learning procedure used for finding them. Experiments and results follow, providing details about the dataset, the performance evaluation measures, and experimental results. Last, we summarize our findings and outline future directions.

## Problem formulation

As is commonly done in the context of classification, and protein-location classification in particular [26,39,40], we represent each protein, *P*, as a weighted feature vector, ${\vec{f}}^{\;P}=\langle )\;f_{1}^{P},\ldots ,f_{d}^{P}\rangle )$, where *d* is the number of features. We view each feature as a random variable *F*_*i*_ representing a characteristic of a protein, such as the presence or absence of a short amino acid motif [5,39], the relative abundance of a certain amino acid as part of amino-acid composition [17], or the annotation by a Gene Ontology (GO) term [41]. Each vector-entry, $f_{i}^{P}$, corresponds to the value taken by feature *F*_*i*_ with respect to protein *P*. In the experiments described here, we use the exact same representation used by Briesemeister et al. [26] as explained in the Experiments and results section, under Data preparation.

We next introduce notation relevant to the representation of a protein’s localization. Let *S*={*s*_1_,…,*s*_*q*_} be the set of *q* possible subcellular components in the cell. For each protein *P*, we represent its location(s) as a vector of 0/1 values indicating the protein’s absence/presence, respectively, in each subcellular component. The *location-indicator vector* for protein *P* is thus a vector of the form: ${\vec{l}}^{\;P}=\langle )l_{1}^{P},\ldots ,l_{q}^{P}\rangle )$ where $l_{i}^{P}=1$ if *P* localizes to *s*_*i*_ and $l_{i}^{P}=0$ otherwise. As with the feature values, each location value, $l_{i}^{P}$, is viewed as the value taken by a random variable, where for each location, *s*_*i*_, the corresponding random variable is denoted by *L*_*i*_. Given a dataset consisting of *m* proteins along with their location vectors, we denote the dataset as: $D=\left\{\right)\left(\right)P_{j},{\vec{l}}^{\;P_{j}}))\;|\;1\leq j\leq m\})$. We thus view the task of protein subcellular multi- location prediction as that of developing a classifier (typically learned from a dataset *D* of proteins whose locations are known) that given a protein *P* outputs a *q*-dimensional location-indicator vector that represents *P*’s localization.

As described in the previous section, most recent approaches that extend location-prediction beyond a single location (e.g. KnowPred_site_[36] and iLoc-Euk [25]), do not consider inter-dependencies among locations. YLoc^+^[26] indirectly considers these inter-dependencies by creating a class for each location-combination. Our underlying hypothesis, which is supported by the experiments and the results presented here, is that directly capturing location inter-dependencies can form the basis for a generalizable approach for location-prediction. We discuss these inter-dependencies next.

Consider a subset of subcellular locations $s_{i_{1}},\ldots ,s_{i_{k}}$. Recall that we use the random variables *L*_*i*_ to denote whether a protein is localized or not to location *s*_*i*_. Formally, the locations in a set, $s_{i_{1}},\ldots ,s_{i_{k}}$, are considered *independent* if for any protein *P*, the joint probability of *P* to be in any of these locations can be written as the product of the individual location probabilities, that is:

$$\text{Pr}\left(L_{i_{1}}=l_{i_{1}}^{P},\ldots ,L_{i_{k}}=l_{i_{k}}^{P}\right)=\overset{k}{\underset{j=1}{\prod }}\mathrm{Pr}\left(L_{i_{j}}=\overset{P}{\underset{i_{j}}{l}}\right).$$

If the locations are *not independent*, that is, if for a protein *P*,

$$\text{Pr}\left(L_{i_{1}}=l_{i_{1}}^{P},\ldots ,L_{i_{k}}=l_{i_{k}}^{P}\right)\neq \overset{k}{\underset{j=1}{\prod }}\mathrm{Pr}\left(L_{i_{j}}=\overset{P}{\underset{i_{j}}{l}}\right),$$

then we say that these locations are *inter-dependent*.

The training of a classifier for protein multi-location prediction involves learning such inter-dependencies so that the classifier can leverage them in the prediction process. We use Bayesian networks to model inter-dependencies.

In order to develop a protein subcellular multi-location predictor, we propose to develop a collection of classifiers, *C*_1_,…,*C*_*q*_, where the classifier *C*_*i*_ is viewed as an “expert” responsible for predicting the 0/1 value, $l_{i}^{P}$, indicating *P*’s non-localization or localization to *s*_*i*_. In order to make use of location inter-dependencies, each *C*_*i*_ uses estimates of location indicators of *P*, ${\hat{l}}_{j}^{P}$ (for all other locations *j*, where *j*≠*i*), along with the feature-values of *P*, in order to calculate a prediction. We use support vector machines (SVMs) (e.g. [20,21]) to compute these estimates. The output of classifier *C*_*i*_ for a protein *P* is given by

(1) $$\;C_{i}(P)=\left\{\begin{array}{c} 1\;\text{If}\;\mathrm{Pr}\left(\overset{P}{\underset{i}{l}}=1\;|\;P,{\hat{l}}_{1}^{P},\ldots ,{\hat{l}}_{i-1}^{P},{\hat{l}}_{i+1}^{P},\ldots ,{\hat{l}}_{q}^{P}\right)>0.5;\\ 0\;\text{Otherwise}. \end{array}\right)$$

Further details about the estimation procedure itself are provided in the Methods sections, under Multiple location prediction.

Bayesian networks have been used before in many biological applications (e.g. [42-44]). In this paper, we use them to model inter-dependencies among subcellular locations, as well as among protein-features and locations. We briefly introduce Bayesian networks here, along with the relevant notations (see [45] for more details). A Bayesian network consists of a directed acyclic graph *G*, whose nodes are random variables, which in our case represent features, denoted *F*_1_,…,*F*_*d*_, and location indicators, denoted *L*_1_,…,*L*_*q*_. We assume here that all the feature values are discrete. To ensure that, we use the recursive minimal entropy partitioning technique presented by Fayyad and Irani [46] and used by Dougherty et al. [47] to discretize the features; this technique was also used in the development of YLoc^+^[26].

**Figure 1:**
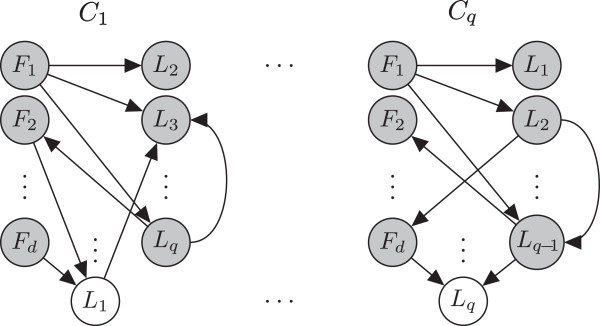
**An example of a collection of Bayesian network classifiers we learn.** The collection consists of several classifiers *C*_1_,…,*C*_*q*_, one for each of the *q* subcellular locations. Directed edges represent dependencies between the connected nodes. There are edges among location variables (*L*_1_,…,*L*_*q*_), as well as between feature variables (*F*_1_,…,*F*_*d*_) and location variables (*L*_1_,…,*L*_*q*_), but not among the feature variables. The latter indicates independencies among features, as well as conditional independencies among features given the locations.

Directed edges in the graph indicate inter-dependencies among the random variables. Thus, as demonstrated in Figure 1, edges are allowed to appear between feature- and location-nodes, as well as between pairs of location-nodes in the graph. Edges between location-nodes directly capture the inter-dependencies among locations. We note that there are no edges between feature-nodes in our model, which reflects an assumption that features are either independent of each other or conditionally independent given the locations. This simplifying assumption helps speed up the process of learning the network structure from the data, while the other allowed inter-dependencies still enable much of the structure of the problem to be captured (as demonstrated in the results). Further details about the learning procedure itself are provided in the Methods section, under Learning Bayesian network classifiers.

To complete the Bayesian network framework, each node *v*∈{*F*_1_,…,*F*_*d*_,*L*_1_,…,*L*_*q*_} in the graph is associated with a conditional probability table, *θ*_*v*_, containing the conditional probabilities of the values the node takes given its parents’ values, Pr(v|Pa(v)). We denote by *Θ* the set of all conditional probability tables, and the Bayesian network is the pair (*G*,*Θ*). A consequence of using the Bayesian network structure is that it represents certain conditional independencies among non-neighboring nodes [45], such that the joint distribution of the set of network variables can be simply calculated as:

(2) $$\;\begin{array}{c} \mathrm{Pr}\left(F_{1},\ldots ,F_{d},L_{1},\ldots ,L_{q}\right)=\overset{d}{\underset{i=1}{\prod }}\mathrm{Pr}\left(F_{i}\;|\;\text{Pa}(F_{i})\right)\\ \;\times \overset{q}{\underset{j=1}{\prod }}\mathrm{Pr}\left(L_{j}\;|\;\text{Pa}(L_{j})\right). \end{array}$$

Figure 1 shows an example of a collection of Bayesian network classifiers. The collection consists of Bayesian network classifiers *C*_1_,…,*C*_*q*_, one for each of the *q* subcellular locations *s*_1_,…,*s*_*q*_, where each classifier *C*_*i*_ consists of the graph *G*_*i*_ and its set of parameters *Θ*_*i*_, (*Θ*_*i*_ not shown in the figure). For each classifier *C*_*i*_, the location indicator variable *L*_*i*_ is the variable we need to predict and is therefore viewed as *unobserved*, and is shown as an unshaded node in the figure. The feature variables *F*_1_,…,*F*_*d*_ are given for each protein and as such are viewed as known or *observed*, shown as shaded nodes in the figure. Finally, the values of the location indicator variables for all locations except for *L*_*i*_, {*L*_1_,…,*L*_*q*_}-{*L*_*i*_}, are needed for calculating the predicted value of *L*_*i*_ in the classifer *C*_*i*_. As such, they are viewed by the classifier as though they are *observed*. Notably, the values of these variables are not known and therefore need to be estimated.

Thus, the structure and parameters of the network for each classifier *C*_*i*_ (learnt as described in the next section), are used to predict the value of each unobserved variable, *L*_*i*_. The task of each classifier *C*_*i*_, is to predict the value of the variable *L*_*i*_ given the values of all other variables *F*_1_,…,*F*_*d*_, and {*L*_1_,…,*L*_*q*_}-{*L*_*i*_}. Since, as noted above, the values of the location indicator variables *L*_*j*_ (*j*≠*i*) are unknown at the point when *L*_*i*_ needs to be calculated, we *estimate* their values, using simple SVM classifiers as described in the Methods section^a^. We note that other methods, such as expectation maximization, can be used to estimate all the hidden parameters, which we shall do in the future.

## Methods

As our goal is to assign (possibly multiple) locations to proteins, we use a collection of Bayesian network classifiers, where each classifier *C*_*i*_, predicts the value (0 or 1) of a single location variable *L*_*i*_ – while using estimates of all the other location variables *L*_*j*_ (*j*≠*i*), which are assumed to be known, as far as the classifier *C*_*i*_ is concerned. The estimates of the location values *L*_*j*_ are calculated using SVM classifiers as described later in this section. The individual predictions from all the classifiers are then combined to produce a multi-location prediction. For each location *s*_*i*_, a Bayesian network classifier *C*_*i*_ must be learned from the training data before it can be used. As described in the previous section, each classifier *C*_*i*_ consists of a graph structure *G*_*i*_ and a set of conditional probability parameters, *Θ*_*i*_, that is: *C*_*i*_=(*G*_*i*_,*Θ*_*i*_). Thus, our first task is to learn the individual classifiers, i.e. their respective Bayesian network structures and parameters. The individual networks can then be used to predict whether a protein localizes to each location.

Given a protein *P*, each classifier *C*_*i*_ needs to accurately predict the location indicator value $l_{i}^{P}$, given the feature-values of *P* and estimates of all the other location indicator values ${\hat{l}}_{j}^{P}$ (where *j*≠*i*). That is, each classifier *C*_*i*_ in the collection assumes that the estimates of the location-indicator values, ${\hat{l}}_{j}^{P}$ for all other locations *s*_*j*_ (where *j*≠*i*) are already known, and is responsible for predicting only the indicator value $l_{i}^{P}$ for location *s*_*i*_, given all the other indicator values. For a Bayesian network classifier this means calculating the conditional probability

(3) $$\begin{array}{l} \mathrm{Pr}(l_{i}^{P}=1\;|\;P,{\hat{l}}_{1}^{P},\ldots ,{\hat{l}}_{i-1}^{P},{\hat{l}}_{i+1}^{P},\ldots ,{\hat{l}}_{q}^{P}), \end{array}$$

under classifier *C*_*i*_, where ${\hat{l}}_{1}^{P},\ldots ,{\hat{l}}_{i-1}^{P},{\hat{l}}_{i+1}^{P},\ldots ,{\hat{l}}_{q}^{P}$ are all estimated using simple SVM classifiers. The classifiers *C*_1_,…,*C*_*q*_ are each learned by directly optimizing an objective function that is based on such conditional probabilities, calculated with respect to the training data.

The procedures used for learning the Bayesian network classifiers and to combine the individual network predictions are described throughout the rest of this section.

### Learning Bayesian network classifiers

Given a dataset *D*, consisting of a set of *m* proteins {*P*_1_,…,*P*_*m*_} and their respective location vectors $\left\{{\vec{l}}^{P_{1}},\ldots ,{\vec{l}}^{P_{m}}\right\}$, each classifier *C*_*i*_ is trained so as to produce the “best” prediction possible for the value of the location indicator $l_{i}^{P}$ (for location *s*_*i*_), for any given protein *P* and a set of estimates of location indicators for all other locations (as shown in Equation 3 above). Based on this aim and on the available training data, we use the *Conditional Log Likelihood (CLL)* as the objective function to be optimized when learning each classifier *C*_*i*_. Classifiers whose structures were learnt by optimizing this objective function were found to perform better than classifiers that used other structures [38]. This objective function is defined as:

$$\;\begin{array}{c} \text{CLL}(C_{i}\;|\;D)\\ \;=\sum_{j=1}^{m}\log\mathrm{Pr}\left(L_{i}=l_{i}^{P_{j}}\;|\;{\vec{f}}^{P_{j}},{{\hat{l}}^{P_{j}}}_{1},\ldots ,{{\hat{l}}^{P_{j}}}_{i-1},{{\hat{l}}^{P_{j}}}_{i+1},\ldots ,{{\hat{l}}^{P_{j}}}_{q}\right). \end{array}$$

Each *P*_*j*_ is a protein in the training set, and each probability term in the sum is the conditional probability of protein *P*_*j*_ to have the indicator value $l_{i}^{P_{j}}$ (for location *s*_*i*_), given its feature vector ${\vec{f}}^{P_{j}}$ and the current estimates for all the other location indicators ${\hat{l}}_{k}^{P_{j}}$ (where *k*≠*i*), under the Bayesian network structure *G*_*i*_ for the classifier *C*_*i*_ (see Equation 2).

**Figure 2:**
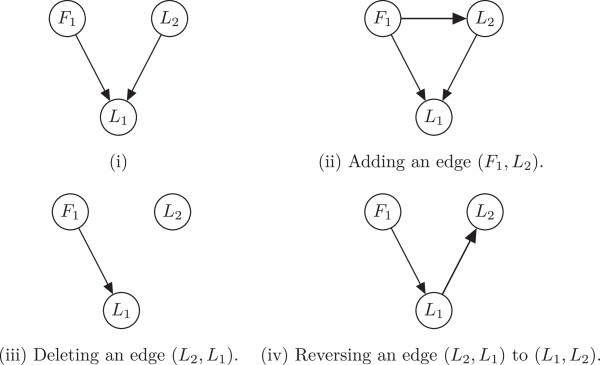
**Adding, deleting, and reversing an edge in a Bayesian network during structure learning.** The network on the left (i), is the starting point. Networks (ii), (iii), and (iv) show the addition, deletion, and reversal of an edge, respectively, as performed by the greedy hill climbing algorithm for structure learning.

To learn a Bayesian network classifier that optimizes this objective function, we use a greedy hill climbing search. While Grossman and Domingos [38] proposed a heuristic method that modifies the basic search depicted by Heckerman et al. [48], we do not employ it in this preliminary study, but rather use the basic search, as the latter does not prove to be prohibitively time consuming. Our structure learner starts with an initial network with no directed edges. In each iteration of the hill climbing algorithm, a directed edge is either added, deleted, or its direction reversed. An example of each of the possible steps is shown in Figure 2. Notably, we do not allow the introduction of directed edges that connect two feature variables to one another. This constraint accounts for the assumption incorporated into the network structure, as discussed in the Problem formulation section, of independence or conditional independence among the features given the locations; it slightly simplifies the network structure and reduces the search space and the overall learning time.

To find estimates for the location indicator values ${\hat{l}}_{k}^{P_{j}}$, we compute a one-time estimate for each indicator $l_{i}^{P_{j}}$ from the feature-values of the protein ${\vec{f}}^{P_{j}}$ by using an SVM classifier (e.g. [20,21]). We employ *q* SVM classifiers, *SVM*_1_,…,*SVM*_*q*_, where each SVM classifier, *SVM*_*i*_ is trained to distinguish a single location indicator *l*_*i*_ from the rest. We use the SVM implementation provided by the Scikit-learn library [49] with a Radial Basis Function kernel. The rest of the network parameters are estimated as follows:

***Parameter learning:*** For each Bayesian network classifier *C*_*i*_, we use the maximum likelihood estimates calculated from frequency counts in the training dataset, *D*, to estimate the network parameters. For each node *v* in the graph *G*_*i*_, (where *v* may either be a feature variable or a location variable), we denote its *n* parents as *Pa*(*v*)={*Pa*_1_(*v*),…,*Pa*_*n*_(*v*)}. For each value *x* of *v* and values *y*_1_,…,*y*_*n*_ of its respective parents, the conditional probability parameter $\mathrm{Pr}(v=x\;|\;{\text{Pa}}_{1}(v)=y_{1},\ldots ,{\text{Pa}}_{n}(v)=y_{n})$ is computed as follows: Let *n*_*joint*_ be the number of proteins in the dataset *D* for whom the value of variable *v* is *x* and the values of *Pa*_1_(*v*),…,*Pa*_*n*_(*v*) are *y*_1_,…,*y*_*n*_, respectively; Let *n*_*marginal*_ be the number of proteins in the dataset *D* whose values of the variables denoted by *Pa*_1_(*v*),…,*Pa*_*n*_(*v*) are *y*_1_,…,*y*_*n*_ (regardless of the value of variable *v*). The maximum likelihood estimate for the conditional probability is thus:

$$\mathrm{Pr}(v=x\;|\;{\text{Pa}}_{1}(v)=y_{1},\ldots ,{\text{Pa}}_{n}(v)=y_{n})=\frac{n_{\text{joint}}}{n_{\text{marginal}}}.$$

To avoid overfitting of the parameters, we add pseudo-counts to events that have zero counts (a variation on Laplace smoothing [50]).

To summarize, at the end of the learning process we have *q* Bayesian network classifiers, *C*_1_,…,*C*_*q*_, like the ones depicted in Figure 1 (one for each of the *q* locations), and *q* SVMs, *SVM*_1_,…,*SVM*_*q*_, used for obtaining initial estimates for each location variable for any given protein. We next describe how these classifiers are used to predict the multi-location of a protein *P*.

### Multiple location prediction

**Figure 3:**
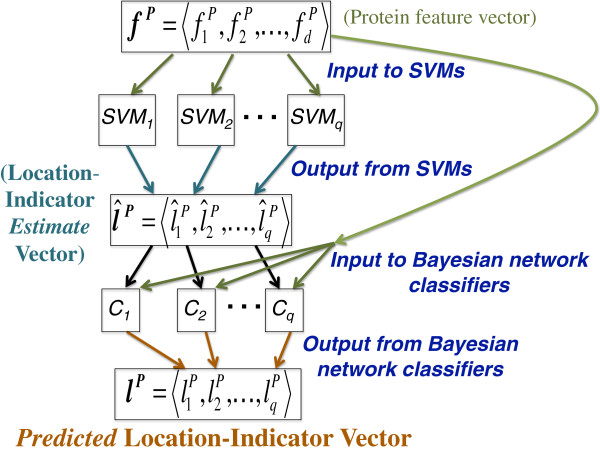
**Multiple location prediction for protein*P*.** First, SVMs *SVM*_1_,…,*SVM*_*q*_ are used to obtain the location indicator estimates ${\hat{l}}_{1}^{P},\ldots ,{\hat{l}}_{q}^{P}$. The Bayesian network classifiers *C*_1_,…,*C*_*q*_ are then used to predict the actual location indicators $l_{1}^{P},\ldots ,l_{q}^{P}$. The Bayesian network classifiers use the location-indicator estimates as well as with inter-dependencies among the locations.

Given a protein *P*, whose locations we would like to predict, we first use the SVMs to obtain preliminary estimates for each of its location indicator values ${\hat{l}}_{1}^{P},\ldots ,{\hat{l}}_{q}^{P}$. We then use each of the learned classifiers *C*_*i*_, and the preliminary values obtained from the SVMs to predict the value of the location indicator $l_{i}^{P}$. The classifier outputs a value of either a 0 or a 1 by thresholding, as shown in Equation 5. The entire process is depicted in Figure 3. The conditional probability of $l_{i}^{P}$ given the feature-values of the protein *P* and the estimates of the location indicator values ${\hat{l}}_{j}^{P}$ (where *j*≠*i*) is first calculated as:

(4) $$\;\begin{array}{c} \mathrm{Pr}\left(\overset{P}{\underset{i}{l}}=1|\;\vec{f^{P}},{\hat{l}}_{1}^{P},\ldots ,{\hat{l}}_{i-1}^{P},{\hat{l}}_{i+1}^{P},\ldots ,{\hat{l}}_{q}^{P}\right)=\\ \;\;\frac{\mathrm{Pr}\left(l_{i}^{P}=1,\vec{f^{P}},{\hat{l}}_{1}^{P},\ldots ,{\hat{l}}_{i-1}^{P},{\hat{l}}_{i+1}^{P},\ldots ,{\hat{l}}_{q}^{P}\right)}{\underset{z\in \{0,1\}}{\sum }\mathrm{Pr}\left(l_{i}^{P}=z,\vec{f^{P}},{\hat{l}}_{1}^{P},\ldots ,{\hat{l}}_{i-1}^{P},{\hat{l}}_{i+1}^{P},\ldots ,{\hat{l}}_{q}^{P}\right)}. \end{array}$$

The joint probabilities in the numerator and the denominator of Equation 4 above are factorized into conditional probabilities using the Bayesian network structure, *G*_*i*_ (see Equation 2). The 0/1 prediction for each $l_{i}^{P}$ obtained from each *C*_*i*_ becomes the value of the *i*’th position in the location-indicator vector $\langle )l_{1}^{P},\ldots ,l_{q}^{P}\rangle )$ for protein *P*. This is the complete multi-location prediction for protein *P*.

In the next section, we describe our experiments using the Bayesian network framework for predicting protein multi-location and the results obtained.

## Experiments and results

We implemented our algorithms for learning and using a collection of Bayesian network classifiers as described above using Python and the machine learning library Scikit-learn [49]. We have applied it to a dataset containing single- and multi-localized proteins, previously used for training YLoc^+^[26]. Below we describe the dataset, the experiments, the evaluation methods we use, and the multiple location prediction results obtained on the proteins from this dataset.

### Data preparation

In our experiments we use a dataset containing 5447 single-localized proteins (originally published as part of the Höglund dataset [39]) and 3056 multi-localized proteins (originally published as part of the DBMLoc set [11] that is no longer publicly available). The combined dataset was constructed and previously used by Briesemeister et al. [26] in their extensive comparison of multi-localization prediction systems. Notably, the protein sequences from the Höglund dataset share no more than 30% sequence identity with each other, while sequences from the DBMLoc dataset share less than 80% sequence similarity with each other. We report results obtained over the multi-localized proteins for comparing our system to other published systems, since the results for these systems are only available for this subset [26]. For all other experiments described here, we report results obtained over the combined set of single- and multi-localized proteins. The single-localized proteins are from the following locations (abbreviations and number of proteins per location are given in parentheses): cytoplasm (*cyt*, 1411 proteins); endoplasmic reticulum (*ER*, 198), extra cellular space (*ex*, 843), golgi apparatus (*gol*, 150), lysosome (*lys*, 103), mitochondrion (*mi*, 510), nucleus (*nuc*, 837), membrane (*mem*, 1238), and peroxisome (*per*, 157). The multi-localized proteins are from the following pairs of locations: cyt_nuc (1882 proteins), ex_mem (334), cyt_mem (252), cyt_mi (240), nuc_mi (120), ER_ex (115), and ex_nuc (113). Note that all the multi-location subsets used have over 100 representative proteins.

#### *Protein representation*

We use the exact same representation of a 30-dimensional feature vector as used by Briesemeister et al. for YLoc^+^[26,51], described below. However, as described later, we also run experiments in which we do not use annotation-based features (items iii and iv in the list below) in the protein representation. 

(i) Thirteen features derived directly from the protein sequence data, specifically, length of the amino acid chain, length of the longest very hydrophobic region, respective number of Methionine, Asparagine, and Tryptophane, occurring in the N-terminus, number of small amino acids occurring in the N-terminus, and numerical values based on: (a) ER retention signal, (b) peroxisomal targeting signal, (c) clusters of consecutive Leucines occurring in the N-terminus, (d) secretory pathway sorting signal, (e) putative mitochondrial sorting signal;

(ii) Nine features contructed using pseudo-amino acid composition [52], which are based on certain physical and chemical properties of amino acid subsequences;

(iii) Two *annotation-based* features constructed using two distinct groups of PROSITE patterns, one characteristic of plasma-membrane proteins and the other of nucleus proteins. For each protein, the value of the respective feature is 1 if the protein sequence contains at least one PROSITE pattern characteristic of the organelle, 0 otherwise;

(iv) Six *annotation-based* features based on GO-annotations. Five of these correspond to five location-specific GO terms [GO:0005783 (endoplasmic reticulum), GO:0005739 (mitochondrion), GO:0005576 (extracellular region), GO:0042025 (host cell nucleus), and GO:0005778 (peroxisomal membrane)], where the feature value is 1 if at least one sequence homologous to the protein’s is associated with the GO term according to Swiss-Prot (release 42.0), 0 otherwise. The sixth feature indicates the likely location of the protein given all the GO terms assigned to it (or to its homologues) in Swiss-Prot;

(See Briesemeister et al. [26,51] for further details regarding the pre-processing, feature construction, and feature selection.)

#### *Feature discretization*

To ensure that all feature values are discrete, we use the minimal entropy partitioning technique as initially presented by Fayyad and Irani [46] and used by Dougherty et al. [47]. We rephrase the partitioning technique by using concepts from Information Theory, in particular, the definition of *conditional entropy*[53]. Each continuous-valued feature is converted into a discrete-valued feature by recursively dividing the range of values that the feature obtains into intervals; all feature values lying within an interval are mapped to a single discrete feature value.

Formally, for a training set of *m* proteins associated with *q* locations *s*_1_,…,*s*_*q*_, we denote the range of values assigned to feature *f*_*i*_ for proteins in the set by $\left[\;l_{f_{i}},h_{f_{i}}\right]$, where $l_{f_{i}}$ is the lowest value in the range and $h_{f_{i}}$ the highest. A *discretization boundaryT*_*i*_ partitions the feature value range $\left[\;l_{f_{i}},h_{f_{i}}\right]$ into two intervals, $\left[\;l_{f_{i}},T_{i}\right]$ and $\left(T_{i},h_{f_{i}}\right]$. For each protein *P*_*j*_ in the set (where 1≤*j*≤*m*), its feature value for feature *f*_*i*_, denoted $f_{i}^{\;j}$, is mapped to a value *d*_1_ if $f_{i}^{\;j}\in [\;l_{f_{i}},T_{i}]$ and to another value *d*_2_ if $f_{i}^{\;j}\in (T_{i},h_{f_{i}}]$, where *d*_1_ and *d*_2_ are two distinct values, chosen from the set {0,1,2,…} (e.g. *d*_1_=0 and *d*_2_=1).

Each location *s*_*k*_ (1≤*k*≤*q*), with which a protein *P*_*j*_ (whose feature value for *f*_*i*_ is $f_{i}^{\;j}$) may be associated, is viewed as a value taken by a random variable *S*. The conditional probability distribution of *S* given a feature value $f_{i}^{\;j}$ and the discretization boundary *T*_*i*_ is defined as:

(5) $$\mathrm{Pr}(S|\;f_{i}^{j},T_{i})=\left\{\begin{array}{cc} \mathrm{Pr}\left(\right)S|\;f_{i}^{j}\leq T_{i})) & \;\;\text{if}\;\;f_{i}^{\;j}\leq T_{i};\\ \mathrm{Pr}\left(\right)S|\;f_{i}^{j}>T_{i})) & \;\;\text{if}\;\;f_{i}^{\;j}>T_{i}. \end{array}\right)$$

The respective conditional entropy is denoted $H\left(\right)S|\;f_{i}^{\;j},T_{i}))$[53] and defined as:

$$\begin{array}{c} H\left(S|\;f_{i}^{\;j},T_{i}\right)=-\mathrm{Pr}\left(f_{i}^{j}\leq T_{i}\right)\overset{q}{\underset{k=1}{\sum }}\left[\right)\mathrm{Pr}\left(S=s_{k}|\;f_{i}^{j}\leq T_{i}\right)\\ \;\times {\text{log}}_{2}\left(\mathrm{Pr}\left(S=s_{k}|\;f_{i}^{j}\leq T_{i}\right)\right)])\\ \;-\mathrm{Pr}\left(f_{i}^{j}>T_{i}\right)\overset{q}{\underset{k=1}{\sum }}\left[\right)\mathrm{Pr}\left(S=s_{k}|\;f_{i}^{j}>T_{i}\right)\\ \;\times \log_{2}\left(\mathrm{Pr}\left(S=s_{k}|\;f_{i}^{j}>T_{i}\right)\right)]), \end{array}$$

where $\mathrm{Pr}(f_{i}^{j}\leq T_{i})$ is estimated as the proportion of proteins in the training set whose feature value for *f*_*i*_ is less than or equal to *T*_*i*_, $\mathrm{Pr}(f_{i}^{j}>T_{i})$ is estimated as the proportion of proteins whose feature value for *f*_*i*_ is greater than *T*_*i*_, $\mathrm{Pr}(s_{k}|\;f_{i}^{\;j}\leq T_{i})$ is estimated by the proportion of proteins associated with location *s*_*k*_ among those whose feature value for *f*_*i*_ is less than or equal to *T*_*i*_, and $\mathrm{Pr}(s_{k}|\;f_{i}^{\;j}>T_{i})$ is estimated by the proportion associated with *s*_*k*_ among those proteins whose feature value for *f*_*i*_ is greater than *T*_*i*_. The discretization boundary *T*_*i*_ is chosen such that the conditional entropy $H(S|\;f_{i}^{\;j},T_{i})$ is minimal.

The partitioning into intervals is applied recursively, and terminates when a stopping condition based on the *Minimum Description Length Principle*, (see Fayyad and Irani [46] for details), is satisfied. This recursive partitioning is independently applied to each of the features.

#### *Exclusion of annotation-based features*

It has been shown by several groups [8,41,54] that protein subcellular location prediction performance is improved by incorporating features based on GO-annotations associated with each protein (which may also include location annotation) into the protein representation. However, we note that an important goal of protein location prediction is to assign locations to proteins that are not yet annotated; that is, the location-prediction tool may serve as an aid in the protein annotation process. Therefore, it is useful to be able to accurately predict location of proteins even without using annotation-based features such as PROSITE patterns and GO terms. To test the performance of our system with and without such features, we have constructed several versions of the dataset in which we include/exclude PROSITE-based and GO-based features. (i) *PROSITE-GO —* which includes both PROSITE- and GO-based features in the protein representation; (ii) *No-PROSITE-GO —* which does not include any PROSITE- or GO-based features in the protein representation; (iii) *No-PROSITE —* which does not include PROSITE-based features, but *includes* GO-based features; and (iv) *No-GO —* which does not include any GO-based features, but *includes* PROSITE-based features, in the protein representation. These datasets are used later in this section (see Classification results) to demonstrate that location inter-dependencies can be used to improve prediction performance, even in the absence of PROSITE-based and GO-based features.

### Experimental setting and performance measures

To compare the performance of our system to that of other systems (YLoc^+^[26], Euk-mPLoc [24], WoLF PSORT [23], and KnowPred_site_[36]), whose performance on a large set of multi-localized proteins was described in a previously published comprehensive study [26], we use the exact same dataset, employing the commonly used stratified 5-fold cross-validation. As the information about the exact 5-way splits used in previous studies is not available, we ran five complete runs of 5-fold-cross-validation (i.e. 25 runs in total), where each complete run of 5-fold cross-validation uses a different 5-way split. The use of multiple runs with different splits helps validate the stability and the statistical significance of the results. To ensure that the results obtained by using our 5-way splits for cross-validation can be fairly compared with those reported before [26], we replicated the YLoc^+^ runs using our 5-way splits, and obtained results that closely match those originally reported by Briestmeister et al [26]. (The replicated *F*_1_-*label* score is 0.69 with standard deviation ±0.01, compared to YLoc^+^ reported *F*_1_-*label* score of 0.68, and the replicated accuracy is 0.65 with standard deviation ±0.01, compared to YLoc^+^ reported accuracy of 0.64). The total training time for our system is about 11 hours (wall-clock), when running on a standard Dell Poweredge machine with 32 AMD Opteron 6276 processors. Notably, no optimization or heuristics for improving run time were employed, as this is a one-time training. For the experiments described here, we ran 25 training experiments, through 5 times 5-fold cross validation, where the total run time was about 75 hours (wall clock).

We use in our evaluation the *adapted* measures of *accuracy* and *F*_1_*score* proposed by Tsoumakas et al. [55] for evaluating multi-label classification. Some of these measures have also been previously used for multi-location evaluation [26,37]. To formally define these measures, let *D* be a dataset containing *m* proteins. For a given protein *P*, let $M^{P}=\left\{\right)s_{i}\;|\;l_{i}^{P}=1$, where 1≤*i*≤*q*} be the set of locations to which protein *P* localizes according to the dataset, and let ${\hat{M}}^{P}=\left\{\right)s_{i}\;|\;{\hat{l}}_{i}^{P}=1$, where 1≤*i*≤*q*} be the set of locations that a classifier predicts for protein *P*, where ${\hat{l}}_{i}^{P}$ is the 0/1 prediction obtained (as described in the Methods section). The multi-label accuracy and the multi-label *F*_1_ score are defined as:

$$\begin{array}{c} \text{Acc}=\frac{1}{\left|D\right|}\underset{P\in D}{\sum }\frac{\left|M^{P}\cap {\hat{M}}^{P}\right|}{\left|M^{P}\cup {\hat{M}}^{P}\right|}\;\text{and}\\ F_{1}=\frac{1}{\left|D\right|}\underset{P\in D}{\sum }\frac{2|M^{P}\cap {\hat{M}}^{P}|}{\left|M^{P}|+|{\hat{M}}^{P}\right|},\;\text{respectively.} \end{array}$$

To evaluate how well our system classifies proteins as localized or not localized to each individual location *s*_*i*_, we use *adapted* measures of multi-label precision and recall denoted ${\text{Pre}}_{s_{i}}$ and ${\text{Rec}}_{s_{i}}$ and defined as follows [26]:

$$\begin{array}{l} {\text{Pre}}_{s_{i}}=\frac{1}{\left|\{P\in D|s_{i}\in {\hat{M}}^{P}\}\right|}\;\;\underset{P\in D|s_{i}\in {\hat{M}}^{P}}{\sum }\frac{\left|M^{P}\cap {\hat{M}}^{P}\right|}{\left|{\hat{M}}^{P}\right|};\\ {\text{Rec}}_{s_{i}}=\frac{1}{\left|\{P\in D|s_{i}\in M^{P}\}\right|}\;\;\underset{P\in D|s_{i}\in M^{P}}{\sum }\frac{\left|M^{P}\cap {\hat{M}}^{P}\right|}{\left|M^{P}\right|}. \end{array}$$

We use here the terms *Multilabel-Precision* and *Multilabel-Recall* to refer to ${\text{Pre}}_{s_{i}}$ and ${\text{Rec}}_{s_{i}}$, respectively. Note that ${\text{Pre}}_{s_{i}}$ captures the ratio of the number of correctly predicted multiple locations to the total number of multiple locations *predicted*, and ${\text{Rec}}_{s_{i}}$ captures the ratio of the number of correctly predicted multiple locations to the number of *original* multiple locations, for all the proteins that co-localize to location *s*_*i*_. Therefore, high values of these measures for proteins that co-localize to the location *s*_*i*_ indicate that the sets of predicted locations that include location *s*_*i*_ are predicted correctly.

Additionally, the *F*_1_-*label* score used by Briesemeister et al. [26] to evaluate the performance of multi-location predictors is computed as:

$$\begin{array}{l} F_{1}\text{-label}=\frac{1}{\left|S\right|}\underset{s_{i}\in S}{\sum }\frac{2\times {\text{Pre}}_{s_{i}}\times {\text{Rec}}_{s_{i}}}{{\text{Pre}}_{s_{i}}+{\text{Rec}}_{s_{i}}}. \end{array}$$

Finally, to evaluate the correctness of predictions made for each location *s*_*i*_, we use the *standard precision* and *recall* measures, denoted by *Pre*-${\text{Std}}_{s_{i}}$ and *Rec*-${\text{Std}}_{s_{i}}$ (e.g. [7]) and defined as:

$$\begin{array}{l} \text{Pre}-{\text{Std}}_{s_{i}}=\frac{\text{TP}}{\text{TP}+\text{FP}}\;\;\text{and}\;\;\text{Rec}-{\text{Std}}_{s_{i}}=\frac{\text{TP}}{\text{TP}+\text{FN}}, \end{array}$$

where *TP* (*true positives*) denotes the number of proteins that localize to *s*_*i*_ and are predicted to localize to *s*_*i*_, *FP* (*false positives*) denotes the number of proteins that do not localize to *s*_*i*_ but are predicted to localize to *s*_*i*_, and *FN* (*false negatives*) denotes the number of proteins that localize to *s*_*i*_ but are not predicted to localize to *s*_*i*_.

### Classification results

Table 1 shows the *F*_1_-*label* score and the accuracy of our system obtained when running over the PROSITE-GO version of the dataset (which includes both PROSITE- and GO-based features in the protein representation), in comparison to those obtained by other predictors (as reported by Briesemeister et al. [26], *Table Three* there), using the same *set of multi-localized proteins* and evaluation measures. While the table shows that our system has a slightly lower performance than YLoc^+^, the differences in the values are not statistically significant (as indicated by the standard deviations of the scores obtained by our system), and the overall performance level is comparable. Thus our approach performs as effectively as current top-systems, while having the advantage of directly capturing inter-dependencies among locations in a generalizable manner (that is, without introducing a new location-class for each new location-combination).

**Table 1:** **Multi-location prediction results on the PROSITE-GO version of the dataset, averaged over 25 runs of 5-fold cross-validation, for multi-localized proteins only, using our system, YLoc**^**+**^**[**[26]**], Euk-mPLoc [**[24]**], WoLF PSORT [**[23]**], and KnowPred**_**site**_** [**[36]**]**

	**Our system**	**YLoc**^**+**^	**Euk-mPLoc**	**WoLF**	**KnowPred**_**site**_
		**[**[26]**]**	**[**[24]**]**	**PSORT [**[23]**]**	**[**[36]**]**
***F***_**1**_**-*label***	0.66 (± 0.02)	0.68	0.44	0.53	0.66
***Acc***	0.63 (± 0.01)	0.64	0.41	0.43	0.63

The *F*_1_-*label* score and *Acc* measures shown for all the systems except for ours are taken directly from *Table Three* in the paper by Briesemeister et al. [26]. Standard deviations are provided for our system (not available for others).

Tables 2 and 3 both show the *F*_1_ score, the *F*_1_-*label* score, and the accuracy obtained by the SVM classifiers (used for computing estimates of location indicators) without using location inter-dependencies, compared with the corresponding values obtained by our system using location inter-dependencies, on the *combined dataset of both single- and multi-localized proteins*. Table 2 displays the scores obtained when running over the PROSITE-GO version of the dataset, whereas Table 3 displays the scores obtained when running over the No-PROSITE-GO, No-PROSITE, and No-GO versions of the dataset (which do not include the respective annotation-based features in the protein representation). All the scores in Tables 2 and 3 obtained using inter-dependencies are higher (in some cases statistically significantly) than those obtained by using SVMs alone without utilizing inter-dependencies. The differences are highly statistically significant (*p*≪0.001), as measured by the 2-sample t-test [56] when running over the PROSITE-GO, No-PROSITE, and No-GO versions of the dataset.

**Table 2:** Multi-location prediction results on the PROSITE-GO version of the dataset, averaged over 25 runs of 5-fold cross-validation, for the combined set of single- and multi-localized proteins, using our system

	***F***_**1**_	***F***_**1**_**-*label***	***Acc***
**SVMs (without using**	0.77 (± 0.01)	0.67 (± 0.02)	0.72 (± 0.01)
**dependencies)**			
**Our system (using**	0.81 (± 0.01)	0.76 (± 0.02)	0.76 (± 0.01)
**dependencies)**			

The table shows the *F*_1_ score, the *F*_1_-*label* score, and the overall accuracy (*Acc*) obtained from SVMs without using location inter-dependencies and from our system, which uses location inter-dependencies. Standard deviations are shown in parentheses.

**Table 3:** Multi-location prediction results on the No-PROSITE-GO, No-PROSITE, and No-GO versions of the dataset, averaged over 25 runs of 5-fold cross-validation, for the combined set of single- and multi-localized proteins, using our system

	**Dataset**	***F***_**1**_	***F***_**1**_**-*label***	***Acc***
**SVMs (without using dependencies)**	No-PROSITE-GO	0.75 (± 0.04)	0.66 (± 0.02)	0.70 (± 0.04)
**Our system (using dependencies)**	No-PROSITE-GO	0.78 (± 0.05)	0.72 (± 0.07)	0.73 (± 0.05)
**SVMs (without using dependencies)**	No-PROSITE	0.77 (± 0.01)	0.66 (± 0.02)	0.72 (± 0.01)
**Our system (using dependencies)**	No-PROSITE	0.80 (± 0.01)	0.75 (± 0.02)	0.75 (± 0.01)
**SVMs (without using dependencies)**	No-GO	0.76 (± 0.03)	0.67 (± 0.03)	0.71 (± 0.03)
**Our system (using dependencies)**	No-GO	0.79 (± 0.04)	0.72 (± 0.08)	0.74 (± 0.04)

The table shows the *F*_1_ score, the *F*_1_-*label* score, and the overall accuracy (*Acc*) obtained from SVMs without using location inter-dependencies and from our system, which uses location inter-dependencies. Standard deviations are shown in parentheses.

Table 3 shows that location inter-dependencies improve multi-location prediction even when annotation-based features, which utilize PROSITE or GO, are not included in the feature set representing the protein. Furthermore, we see from Tables 2 and 3 that the performance of our system does not deteriorate substantially when running over dataset versions that do not include various annotation-based features. Thus, our system shows robustness to the presence/absence of annotation-based features.

Table 4 shows the prediction results obtained by our system when running over the PROSITE-GO version of the dataset for the five locations that have the largest number of associated proteins: cytoplasm (cyt), extracellular space (ex), nucleus (nu), membrane (mem), and mi (mitochondrion), on the *combined dataset of both single- and multi-localized proteins*. For each location *s*_*i*_, we show the *standard precision* (*Pre*-${\text{Std}}_{s_{i}}$) and *recall* (*Rec*-${\text{Std}}_{s_{i}}$) as well as the *Multilabel-Precision* (${\text{Pre}}_{s_{i}}$) and *Multilabel-Recall* (${\text{Rec}}_{s_{i}}$). The table shows values for each of the measures obtained by SVMs without using location inter-dependencies and by our system using location inter-dependencies. When using inter-dependencies, for a few locations, such as *cytoplasm* and *membrane*, the *Multilabel-Precision* (${\text{Pre}}_{s_{i}}$) decreases. Nevertheless, most of the differences are not highly statistically significant (*p*>0.01), as measured by the 2-sample t-test [56]. The *Multilabel-Recall* (${\text{Rec}}_{s_{i}}$) increases for all locations with the use of inter-dependencies where the differences in most cases are highly statistically significant (*p*≪0.001). We examine the statistically significant differences in the *Multilabel-Recall* for cytoplasm (3785 proteins), membrane (1824), and peroxisome (157). The *Multilabel-Recall* for cytoplasm (*Rec*_*cyt*_) increases from 0.78 when classifying by SVMs without using inter-dependencies, to 0.80 when incorporating inter-dependencies. The *Multilabel-Recall* for membrane (*Rec*_*mem*_) increases from 0.76 to 0.78 under similar conditions. Even for a location like peroxisome that has fewer associated proteins, the *Multilabel-Recall* increases from 0.37 using simple SVMs to 0.65 using our classifier. Our analysis demonstrates the advantage of using location inter-dependencies for predicting protein locations, not just for locations that have a large number of associated proteins but also for locations that are associated with relatively few proteins.

**Table 4:** Multi-location prediction results on the PROSITE-GO version of the dataset, per location, averaged over 25 runs of 5-fold cross-validation, for the combined set of single- and multi-localized proteins

	**cyt (3785)**	**ex (1405)**	**nuc (2952)**	**mem (1824)**	**mi (870)**
***Pre***-${\text{Std}}_{s_{i}}$**(SVMs)**	**0.84 (± 0.01)**	0.87 (± 0.02)	**0.79 (± 0.02)**	**0.93 (± 0.01)**	**0.90 (± 0.03)**
***Pre***-${\text{Std}}_{s_{i}}$**(Our system)**	**0.84 (± 0.01)**	**0.91 (± 0.02)**	**0.79 (± 0.03)**	0.90 (± 0.01)	0.87 (± 0.03)
***Rec***-${\text{Std}}_{s_{i}}$**(SVMs)**	0.85 (± 0.01)	0.64 (± 0.02)	0.72 (± 0.02)	0.79 (± 0.02)	0.62 (± 0.03)
***Rec***-${\text{Std}}_{s_{i}}$**(Our system)**	**0.86 (± 0.01)**	**0.65 (± 0.02)**	**0.74 (± 0.03)**	**0.80 (± 0.02)**	**0.66 (± 0.03)**
${\text{Pre}}_{s_{i}}$**(SVMs)**	**0.82 (± 0.01)**	0.89 (± 0.02)	**0.83 (± 0.01)**	**0.92 (± 0.01)**	0.87 (± 0.03)
${\text{Pre}}_{s_{i}}$**(Our system)**	0.81 (± 0.02)	**0.91 (± 0.02)**	**0.83 (± 0.01)**	0.90 (± 0.01)	**0.89 (± 0.02)**
${\text{Rec}}_{s_{i}}$**(SVMs)**	0.78 (± 0.01)	0.72 (± 0.02)	0.77 (± 0.01)	0.76 (± 0.01)	0.68 (± 0.02)
${\text{Rec}}_{s_{i}}$**(Our system)**	**0.80 (± 0.01)**	**0.74 (± 0.02)**	**0.78 (± 0.02)**	**0.78 (± 0.01)**	**0.73 (± 0.02)**

Results are shown for the five locations *s*_*i*_ that have the largest number of associated proteins (the number of proteins per location is given in parenthesis): cytoplasm (cyt), extracellular space (ex), nucleus (nuc), membrane (mem), and mitochondrion (mi). The table shows the per-location measures: *standard precision* (*Pre*-${\text{Std}}_{s_{i}}$), *recall* (*Rec*-${\text{Std}}_{s_{i}}$), *Multilabel-Precision* (${\text{Pre}}_{s_{i}}$), and *Multilabel-Recall* (${\text{Rec}}_{s_{i}}$), obtained from SVMs without using location inter-dependencies and from our system using location inter-dependencies. For each location and measure, the highest of the values obtained from the two methods is shown in boldface. Standard deviations are shown in parentheses.

## Discussion and conclusions

We presented a new way to use a collection of Bayesian network classifiers, taking advantage of location inter-dependencies, to provide a generalizable method for predicting possible multiple locations of proteins. The results demonstrate that the performance of our preliminary system is comparable to the current best performing multi-location predictor YLoc^+^[26]. The latter indirectly addresses dependencies by creating a class for each multi-location combination. Our results also show that utilizing inter-dependencies significantly improves the performance of the location prediction system, with respect to SVM classifiers that do not use any inter-dependencies. Moreover, this improved performance due to the use of location inter-dependencies is maintained even when the protein representation does not include PROSITE patterns-based features or GO-based features, thus exhibiting robustness to the presence/absence of annotation-based features.

In most biological applications that have used Bayesian networks so far (e.g. [42-44]), the variable-space typically corresponds to genes or SNPs which is a very large space and necessitates the use of strong simplifying assumptions and many heuristics. In contrast, we note that predicting multiple locations for proteins involves a significantly smaller number of variables (as the number of subcellular components and the number of features for representing proteins are relatively small), making this task ideally suitable for the use of Bayesian networks.

The study presented here is a first investigation into the benefit of directly modeling and using location inter-dependencies. To obtain initial estimates for location values, we used a simple SVM classifier, and location inter-dependencies were only learned based on these values. While the results already show much improvement with respect to the baseline SVM classifiers, we believe that a better approach would be to simultaneously learn a Bayesian network while estimating the location values using iterative optimization methods such as expectation maximization.

We note that although the dataset we use is the most extensive available collection of multi-localized proteins, several subcellular locations are not represented in the dataset at all due to the low number of proteins associated with them. Similarly, there is not enough data pertaining to proteins that are localized to more than two locations. We are in the process of building a set of multi-localized proteins that will be used in future work to test the performance of our system on new, and more complex, combinations. We also plan to explore alternative approaches for learning models of location inter-dependencies from the available data.

## Endnote

^a^ We note that here we set out to show that capturing inter-dependencies among locations help improve prediction, and the relatively simple estimation procedure that we use serves sufficiently well.

## Competing interests

The authors declare that they have no competing interests.

## Authors’ contributions

RS and HS conceived the presented ideas, performed data analysis, and have written the manuscript. RS implemented the methods and performed the experiments. Both authors have read and approved the final manuscript.
